# The Challenging Path to Developing a Mobile Health Device for Epilepsy: The Current Landscape and Where We Go From Here

**DOI:** 10.3389/fneur.2021.740743

**Published:** 2021-10-01

**Authors:** Ilona Hubbard, Sandor Beniczky, Philippe Ryvlin

**Affiliations:** ^1^Department of Clinical Neurosciences, Vaud University Hospital, Lausanne, Switzerland; ^2^Department of Clinical Neurophysiology, Danish Epilepsy Center, Dianalund, Denmark; ^3^Department of Clinical Neurophysiology, Aarhus University Hospital, Aarhus, Denmark

**Keywords:** epilepsy, seizure detection, seizure forecasting, mobile health devices, extracerebral biosensors, wearables, EEG signals, usability and user experience

## Abstract

Seizure detection, and more recently seizure forecasting, represent important avenues of clinical development in epilepsy, promoted by progress in wearable devices and mobile health (mHealth), which might help optimizing seizure control and prevention of seizure-related mortality and morbidity in persons with epilepsy. Yet, very long-term continuous monitoring of seizure-sensitive biosignals in the ambulatory setting presents a number of challenges. We herein provide an overview of these challenges and current technological landscape of mHealth devices for seizure detection. Specifically, we display, which types of sensor modalities and analytical methods are available, and give insight into current clinical practice guidelines, main outcomes of clinical validation studies, and discuss how to evaluate device performance at point-of-care facilities. We then address pitfalls which may arise in patient compliance and the need to design solutions adapted to user experience.

## Introduction

We live in the Internet of Things (IoT) era where wearables have become an integral part of day-to-day life. Health and sleep trackers, fitness wristbands, smartwatches, and other technologies attached to the human body offer users continuous biometric measurements (i.e., movement, heart rate, sweating) over time, providing analytics via Bluetooth (or similar wireless transmission protocols, e.g., wi-fi), and sending feedback to applications integrated into generic devices (e.g., smartwatches). These wearable devices offer considerable potential in regards to delivering novel options to shape personalized medical solutions (1). In the field of epilepsy, specific types of wearable solutions have been developed with the aim of detecting individual seizures, several of which have penetrated the consumer market. The overarching goal of these devices is to provide continuous long-term monitoring of non-EEG seizure related signals in order to detect or forecast seizures (2–9). Currently, however, seizure detection with appropriate sensitivity and false-alarm rates has only been possible for generalized tonic-clonic seizures (GTCS) (10, 11), while clinically-relevant accuracy is still lacking for most other seizures types (12). Solutions to this limitation are likely to emerge from the rapid advances in machine-learning seizure detection and algorithms forecasting the cyclic nature of seizures (13, 14). In parallel, progress has been made in the field of long-term ambulatory EEG (15). Subcutaneous electrode implants, embedded with a wearable data receiver have allowed for the first-time real-world EEG data collections over very long periods (16).

This short review gives an insight into the ambitious and challenging field of wearable health devices for seizure detection and forecasting applications and provides context for progress to date, as well as possible pitfalls and how they might be resolved.

## Clinical Applications

With those suffering from chronic epilepsy, IoT technologies have the potential to advance personalized epilepsy management strategies, with the end result of increasing the positive disease outcome for patients. To date, the epilepsy community has demonstrated significant interest in wearable seizure detection (17–21) and forecasting (22) companions. In the following section, we have highlighted several avenues for their clinical application, which could prove clinically relevant and useful to patients.

In patients with high seizure frequency (20) and GTCS, alarm-triggering to a caregiver's smartphone might prompt life-saving procedures and prevent Sudden Unexpected Death from Epilepsy (SUDEP). It might also help decreasing the risks of SUDEP and of other causes of GTCS-related mortality (23–25) and morbidity, by providing predictive biomarkers of such outcomes (12, 26) and of GTCS severity (26). However, no study has yet demonstrated that the use of mobile health solutions, including seizure detection devices coupled with alarms has an impact on the risk of SUDEP.

A reliable seizure detection device could also provide physicians with more accurate information on seizure frequency than patients' diary entries or recollection, which in turn can help adjust both the type and dosage of medication and subsequent treatment outcome, [e.g., (27–31)]. However, despite long-term ambulatory EEG (32) and/or ambulatory implanted intracranial EEG (15) studies demonstrating that patients tend to under-report seizures, there is still need for more evidence to show that optimized seizure tracking for patients who use connected devices, is more reliable than diaries or direct communication with the patient. For instance, a phase-4 study has shown that a wearable accelerometer based system helped patients and caregiver to log GTCS into the seizure diary in 44% of the cases (32).

Major disruptions to quality of life can be attributed to the uncertainty of when seizures will occur (33). When responding to questions concerning their medical history, patients commonly report a periodicity in seizure occurrence and familiarize themselves with individual risk factors and triggers that they observe preceding seizure events. These then serve as reference points for certain individuals to keep an overview of their disease progression (34–39). The capacity to foresee a seizure event would transform epilepsy care and ultimately allow patients to counteract impending seizures by proactively adapting their behavior. For example, a patient could potentially self-target neurostimulation to abort the seizure (40), or rely on a closed-loop apparatus that offered immediate seizure-triggered therapy (41).

## Sensors

EEG and non-EEG sensors can be embedded in a wearable tool, and are directly in contact with the body in order to acquire physiological signals. The set-up should be appropriate to measure epilepsy-related activity over long periods of time during the day and night and has specific constraints in terms of comfort and stigma (42). Especially for EEG systems, usability comes as a challenge, as full-array scalp electrodes can only be used in hospitals, or in controlled home-monitoring settings (43), over time periods of up to several days maximum. Non-obtrusive solutions have been developed, using only few electrodes positioned either around (44) or in the ear (45, 46), but signal quality often remains inadequate, characterized by artifacts and sub-optimal electrode impedance. In order to facilitate continuous EEG recordings, subcutaneous (a.k.a. subscalp or subdermal) electrodes, placed by means of a minimally invasive surgical procedure under the scalp, have been proposed (37, 47). Subcutaneous electrodes set-up showed similar SNR levels, and even better signal quality than standard scalp EEG montages (48). After the development of first prototypes (49–51), a few products have started their incubation process, but so far, only one device has undergone clinical trials to be approved for commercial use by regulatory bodies (52). First studies in epilepsy patients showed that surgeries (37, 47) and ultra-long-term use for real life monitoring for up to 3 months at home (37) were technically feasible, well-tolerated, and for in a first use-case with eight epilepsy patients, successfully detected seizures (37, 47). Recently, a larger multicenter trial involving the implantation of subcutaneous EEG devices in 14 patients with epilepsy and 12 healthy subjects, demonstrated the technological viability of stable long-term EEG recordings (52).

**Figure 1:**
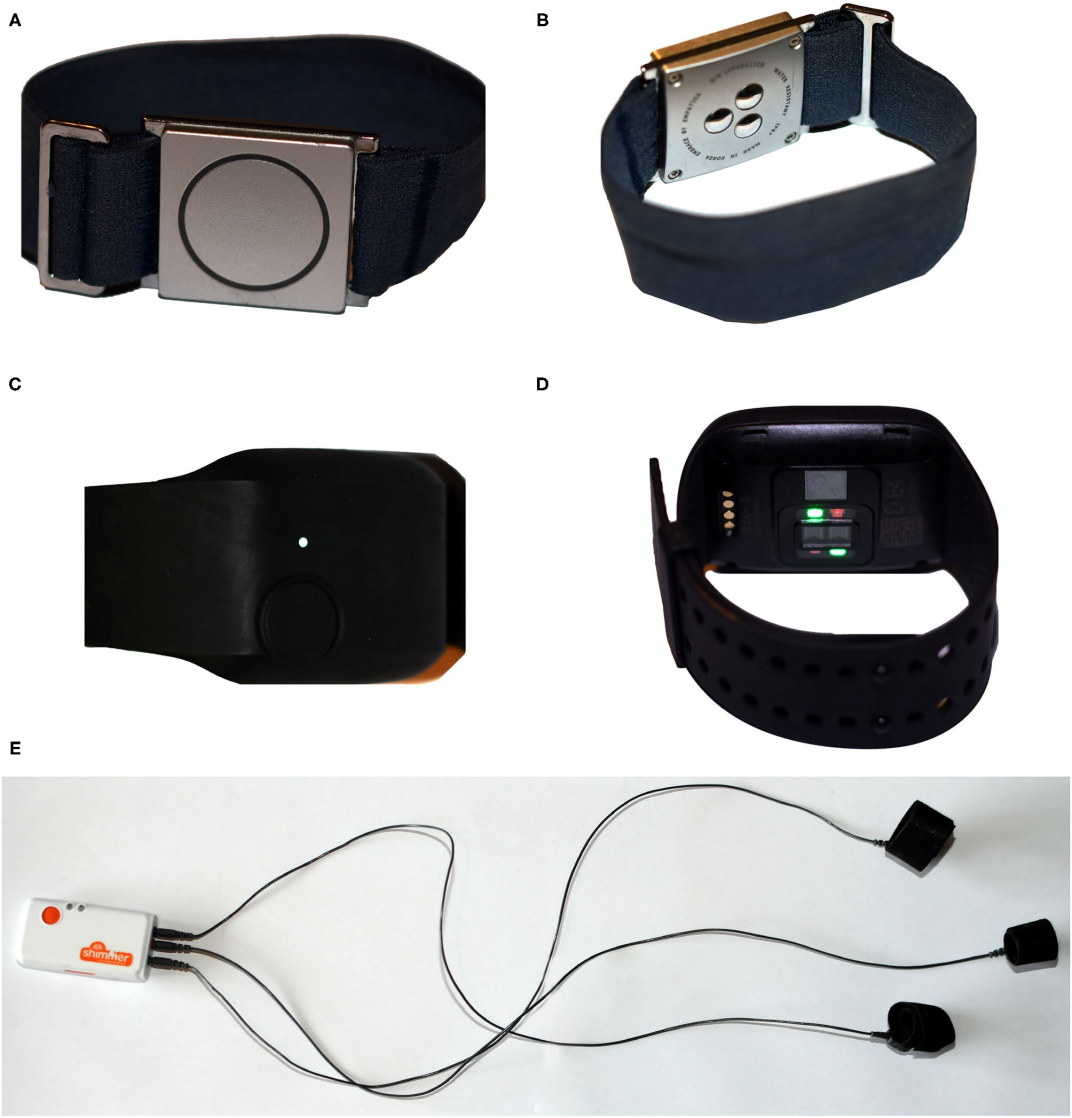
**(A,B)** A wrist worn commercially available seizure detector with integrated ACC and EDA sensor, **(B)** displays three electrodes for EDA measure that are in contact with the skin. **(C,D)** A wrist worn commercially available sensing device with integrated ACC, EDA, and PPG sensor. **(D)** Displays the PPG sensor that is in contact with the skin. **(E)** A wired galvanic skin response (EDA) sensing solution, with velcro fabric finger cuffs and a small receiver unit that attaches to the wrist.

Currently, four additional diagnostic solutions are progressing in their development (16). Different electrode designs (i.e., bipolar electrodes, multichannel strips), and placement underneath the scalp (i.e., from focal to covering both hemispheres) determine the type of seizures that can be recorded (37, 47). Subcutaneous solutions are quasi invisible and existing studies show that patients are willing to undergo the operation and insertion of the material for long periods of time (37, 47, 53). However, additional data involving larger patient samples are required to confirm overall acceptance of these devices. Non-EEG based sensors, are primarily based on accelerometers, surface electromyography (EMG), electrocardiography (ECG), photoplethysmography (PPG) and electrodermal activity (EDA). They are easily integrated in fashionable wearables, such as bracelets, without displaying the disease and stigmatizing patients. Accelerometers strapped to a limb appropriately identify GTCS, and other seizures with strong motor components (54–56). Surface EMG on the biceps muscle is particularly useful for the detection of tonic seizures early on in the course of GTCS (57–60). The prerequisite for performance of movement sensors is to fasten them to a body part that participates in the seizure semiology. Heart rate parameters are known to vary during and even shortly before seizures (61, 62). They can be recorded using self-adhesive ECG electrode patches (63, 64) or via measures of heart rate (HR) by means of an optical sensor that captures a PPG signal at the wrist (65, 66). Finally, a clear-cut surge in EDA is observed at onset of GTCS (67–69), while imbalance in this autonomic biomarker can also be observed in the pre-ictal state (70). A combination of signals can be used for seizure monitoring in the real-world setting, yet the pitfall of this approach is that signal quality, and thus reliability of the approach, is influenced by daily activities and psychological arousal (70, 71). Please view Figures 1A–E for an illustration of different form factors of sensing solutions.

## Possible Analytical Approaches

In validation studies, the accuracy of seizure detection algorithms must be compared to the gold standard for seizure-diagnosis: video-EEG recording or (for motor seizures) video recordings. The presence or absence of an epileptic event will then be mathematically treated as a binary variable. To increase the accuracy of seizure detection algorithms, various machine learning methods are available (72). Their performance is typically evaluated by determining the area under a receiver-operating characteristic curve (ROC) (2–9). Seizure prediction algorithms aim at identifying pre-ictal changes immediately before the seizure in order to anticipate the precise onset of a given seizure (73–77). Meanwhile, data from a clinical trial (15, 36), animal studies (34, 78–81), crowdsourcing analysis efforts of EEG databases (82, 83), and cohort studies of mobile seizure diaries (39, 84, 85), have identified that epileptic activity in some patients followed circadian, ultradian, and infradian (multidian) cycles, and was highly correlated with self-forecasting techniques (86). Thus, an alternative conceptual approach (87) to single seizure event prediction, is the ability to algorithmically forecast them. A successful seizure forecast would provide the patient with a seizure cluster likelihood measure within a given time-window, based on intraindividual cyclic distribution of events. Companions using this approach might require more complex designs than their seizure detection analogs. For instance, the varying scales of forecasting windows (22) and data visualization features (88) would be attuned according to frequency and characteristics of the seizure cycles. Forecasting will also benefit from the development of new EEG methods for very-long-term data collection. Currently, clinical trials have been advised (89) and are urgently needed to further existing knowledge about seizure networks, the characteristics of interictal changes, ictogenesis, as well as their cyclicity and predictive value over prolonged time periods (13, 90, 91). Research on seizure periodicity function will constitute the basis for addressing this bottleneck in the advance of algorithmic approaches.

## Research and Development

In Europe, clinical trials of wearable devices are steadily increasing and are required to follow the regulatory framework of *Council Directive 93/42/EEC* concerning medical devices. In order to optimize study quality in the epilepsy domain, recommendations for standardized testing and clinical validation of seizure detection devices have been recently proposed (92). This new standard classifies studies into 5 phases (0–4), with key trial design features concerning the number of subjects, types of recordings, data analysis, and alarm criteria. For example, whereas a phase 0 study can still be performed on retrospective datasets and serve as a proof of concept for algorithm development, subsequent phases will require technical feasibility testing and signal validation against standard hospital Video-EEG. The final phase (4), will have to include a large patient sample and be performed in a prospective manner at multiple sites under real world-settings. As previously proposed (76), seizure detection criteria for all devices will represent the first qualitative control with the aim of complementing aspects specifically relevant for digital forecasting companions. Overall, device developers must consider that high quality product R&D will require several years. Furthermore, trials will have to contain a clear set of outcome measures and criteria to be reported to and evaluated by regulatory bodies. Result summaries containing transparent research data should also be made available to the medical community at large.

## Device Validity

The working group of the International League Against Epilepsy (ILAE) and the International Federation of Clinical Neurophysiology (IFCN) recently performed a literature search and published evidence from 28 papers (10, 11), leading to consensus in the endorsement of “*clinically validated wearable devices for automated detection of GTCS including the focal-to-bilateral tonic-clonic seizures (FBTCS) when significant safety concerns exist, especially in unsupervised patients (…)*.” Only a handful of validation studies have focused on the detection of focal, non-convulsive seizures (10–12). As a consequence, no commercially available wearable devices have received regulatory authorization for the detection of focal epileptic seizures, and “*the ILAE-IFCN Working Group, does not recommend clinical use of the currently available wearable devices for seizure types other than GTCS and FBTCS, as more research and development are needed for this application (…)*.”

For the purposes of this short review, we have summarized and updated the efforts of the ILAE-IFCN workforce below (10, 11). Table 1 included the outcomes of nineteen phase 2–4 prospective studies that span the development of novel analytical methods, confirm their safety and accuracy with respect to regulatory bodies, and their ability to assess seizure detection performances in the patient's homes, as opposed to the laboratory environment.

**Table 1:** Outcomes of phase 2–4 prospective seizure detection algorithm reliability studies.

**Modality**	**Study**	**Phase**	**Study site/design**	**Seizure type**	**Device**	**Performance**	**Time to positive detection after seizure onset**
Surface EMG	Szabo et al. (59)	2	EMU/Offline	GTCS	Brain Sentinel	Sensitivity = 95% FP = 0.017/24 h	μ = 15.2 s
Surface EMG	Halford et al. (93)	2	EMU/Offline	GTCS	Brain Sentinel	Sensitivity depending on cohort = 100%, 76% FP = 1.4/24 h, 2.5/24 h	μ = 7.45 s
Surface EMG	Beniczky et al. (94)	3	EMU/Real-time	GTCS	Seizure detector by Ictal Care	Sensitivity = 93.8% FP = 0.67/24 h	μ = 9 s
Wrist 3D-ACC	Kramer et al. (95)	2	EMU/Real-time	Motor seizures, GTCS	Not specified	Sensitivity = 90.9% FP = 0.11/24	μ = 17 s
Wrist 3D-ACC	Beniczky et al. (96)	3	EMU/Real-time	GTCS	Epi-care	Sensitivity = 89.7% FP = 0.2/24 h	μ = 33 s from GTCS, μ = 55 s from FS
Wrist 3D-ACC	Meritam et al. (32)	4	In-field/Real-time	GTCS	Epi-care	Sensitivity = 90% FP = 0.1/24 h	n.a (infield)
Wrist ACC	Patterson et al. (97)	2	EMU/Real-time	GTCS, tonic, myoclonic, hypermotor, complex partial	Smartwatch by Smartmonitor	GTCS: Sensitivity = 31% FS: Sensitivity = 16% FP = n.r	Not reported
Wrist ACC	Velez at al. (98)	2	EMU/Real-time	GTCS	Smartwatch by Smartmonitor	GTCS: Sensitivity = 92.3% FP = n.r FS = Not detected	Not reported
Wrist 3D- ACC	Johansson et al. (55)	2	EMU/Offline	GTCS	Shimmer	Sensitivity = 90–100% FP = 0.24–1.2/24 h	Not reported
Wrist ACC + EDA	Onorati et al. (99)	2	EMU/Offline	GTCS	Embrace	Sensitivity = 94.55% FP = 0.2/24 h	Median = 29.3%
ACC +ECG	Van Andel et al. (100)	2	EMU/Offline	GTCS, tonic, clonic, hypermotor	Shimmer	Clinically urgent GTCS: Sensitivity = 87% FS: Sensitivity = 56–71% FP = 2.3–5.7/24 h for all seizures per night	μ = 13 s
Wrist 3D-ACC + PPG	Arends et al. (101)	3+4	EMU + In-field/Real-time	GTCS, myoclonic/ tonic, hyperkinetic	Nightwatch	GTCS: Sensitivity = 81% Other motor seizures: Sensitivity = 77% FP = 0.3 per night	Not reported
ECG	Boon et al. (102)	2	EMU/Offline	GTCS, FS	Hospital ECG& VNS Aspire SR	For HR increase >20%: Sensitivity = 52.3% FP = 7.2/h	Not reported
ECG	Fisher et al. (103)	2	EMU/Offline	GTCS, FS	Hospital ECG&VNS Aspire SR	For HR increase >20%: Sensitivity = 91% FP = 0.7/24 h	μ = 8 s
ECG, PPG	Vandecasteele et al. (104)	2	EMU/Offline	FS	Hospital ECG, 180° eMotion, E4	ECG: Sensitivity = 57% FP = 1.92/24 h 180°: Sensitivity = 70% FP = 2.11/24 h E4: Sensitivity = 32% FP = 1.8/24 h	Not reported
ECG	Jeppesen et al. (63)	2	EMU/Offline	GTCS, FS	ePatch ECG	GTCS: Sensitivity = 100% FS: Sensitivity = 90.5% FP = 1/24 h	μ = 30 s
In-the-ear NIRS	Jeppesen et al. (105)	2	EMU/Offline	FS	PortaLite	Sensitivity = 6–24%	Not reported
Behind-the-Ear-EEG	Gu et al. (106)	2	EMU/Offline	FS	Ambu Neuroline Cup	Sensitivity = 94.5% FP = 0.52/24 h	Not reported
PPG + Oxygen saturation	Brotherstone et al. (107)	3	EMU/Real-time	Clinically significant seizures	Nonin finger sensor	For HR change > 25% + Oxygen desaturation <85%: Sensitivity = 87% FP = 4.5/24 h	μ = 69.6 s for HR μ = 83 s for oxygen desaturation

*EMG, Electromyogramm; EMU, Epilepsy monitoring unit; FP, False positive; GTCS, Generalized tonic-clonic seizures; HR, heart rate; h, hour; μ, mean; n.a, not applicable; NIRS, near-infrared spectroscopy; s, second*.

There is ample evidence regarding the detection rates for convulsive seizures using a self-adhesive EMG patch placed on the bicep muscle. In this case, a reasonable true positive/false positive ratio was achieved using the algorithm, with a GTCS detection sensitivity of 94% and a false alarm rate of 0.7/24-h (94). Interestingly, when different detection-thresholds were used, 100% sensitivity could be attained, however the false positive rate was consequently increased to 1.44/24-h (93).

An alternative GTCS detection method using wrist accelerometers has approached a relatively high sensitivity of >89.7% in several phase 2 studies, with the rate of false alarms significantly reduced <0.24 per day (32, 55, 95–98). ACCs are most effective when attached to several areas of a patient's body known to be implicated in the seizure semiology. Using multiple ACCs, several phase 1 studies, identified myoclonic-, clonic-, tonic- (108, 109), and hypermotor seizures (110, 111) with a sensitivity rate of at least 95%. However, a different phase 2 study found that ACC-based wristwatches were less sensitive for the detection of different types of focal motor seizures (0–24%) (97).

Conversely, heart rate parameters from ECG systems were able to detect arrhythmias associated with focal seizures and were showed sensitivity as high as (<91%). Nonetheless, many algorithms still produced false positives from 0.5 to 5.4 per hour (112–114). PPG signals measured from the wrist, may also be appropriate detectors, however, they have so far not been shown to have lower false positives rates than hospital ECGs (2.11 vs. 1.92) (104).

Combining the outputs from several sensors into a single algorithm, has been considered in order to raise the detection rate of GTCS (21, 115, 116). For instance, a clinical trial combining phase-3 and 4, evaluated a band placed on the upper arm, recording accelerometry and heart rate, and succeeded to isolate nocturnal GTCS with a sensitivity of 81% (and 77% of motor seizures), and a reduced false alarm rate of 0.03 per night (101). A phase 2 EMU study, using a retrospective cross-validation approach, demonstrated that fusing accelerometry signals with electrodermal activity (EDA) recorded from a wrist-worn device, resulted in a sensitivity of 95% for GTCS and a false positive rate of 0.2 per day (99). Most recently, a phase 3 EMU study applied pre-defined cut-off points to data obtained in real-time, and showed that when heart rate changes and oximetry endpoints are combined, the sensitivity is highest, and lowest when the parameters are used alone (117).

Wearable EEG devices are potentially well-suited to reflect the focal seizure onset when placed above the area of seizure onset. A phase two study performed in the EMU showed the aptitude of a behind the-ear-EEG set-up with a sensitivity of 94.5%. In order to allow for ultra-long term monitoring, with the goal to create personalized seizure forecasting (34, 37–39), subcutaneous wearable EEG devices are currently being developed (16). To date, only one device has received approval for sale by the regulatory authorities (52). For this purpose, Duun-Herniksen et al. performed EMU-based clinical trials for safety and signal quality in patients with epilepsy originating from the temporal lobe (47, 48) and performed the first ultra-long-term home study in a sample of eight patients. This real-life monitoring, for up to 3 months, was shown to be safe, well-tolerated by participants, and technically feasible (37).

## Clinical Evidence

While there is strong evidence to prove the accuracy of non-EEG wearables, the average time to detect a seizure has been situated between 7.45 (90) and 83 s (91) from onset. Therefore, until now the purpose of seizure companions has mostly found applications such as alarm systems for SUDEP prevention, and the optimization of seizure diaries. However, first studies have been published that report having successfully developed seizure forecasts based on extracerebral biosignals (76, 118). Phase 4 trials for subcutaneous EEG seizure prediction/forecasting devices have yet to be performed (93). Consequently, clinical evidence on the impact of wearable solutions is sparse, and has so far, only been indirectly deduced from epidemiological data reflecting mortality rates in unsupervised patients who do not share a bedroom with another person and are found in prone position following a GTCS (100).

## User Expectations and First Experiences With Device Implementation

Surveys have demonstrated the clinical relevance of seizure detection, giving patients (22, 119, 120), as well as caregivers and healthcare professionals (17–20, 22), the opportunity to express their needs and wishes for wearable health companions. Several studies have specifically addressed user feedback about acceptable rates in both sensitivity and false alarms (19, 20, 120), finding that at minimum, patients desire both an accurate detection/prediction rate > 90% and incorrect seizure triggering of less than once per day. However, at present only the ACC wristband and EMG patch have achieved this reliability ratio for the detection of GTCS, whereas other available consumer devices have not (121). One way to increase seizure recognition has been to lower the threshold, although consequent higher false positive rates exist, creating a challenge for software developers who must then determine how best to tune their devices. For example if a seizure is missed (false negative), this is considered more harmful than incorrectly identifying it (false positive) (120). Furthermore, in patients where seizure frequency is lower, false alarms were more tolerable, as statistically they would occur only under certain conditions. Nonetheless, interest in continuous real-time monitoring and alarms has increased, especially in patients with a high seizure frequency and concomitant risk of SUDEP (20), where high rates of daily false alarms would become unbearable for those most affected. Nonetheless, it was shown that a system could possibly permit users to tune their sensitivity and false positive rates (122), allowing to adjust for the patients' individual preferences of control, which might be quite different (123).

Regarding seizure forecasting, to date only one survey has directly questioned patient and caretaker preferences, finding that high accuracy and short forecasting windows were favored over long-term, less precise ones (22). Broadly speaking, forecasting has significant potential, as it covers physiological parameters and time-frames of various length, with the ability to provide predictive information of higher complexity than a binary seizure detector (76). To outline future applications of this methodology, further studying of end-user requirements and focusing on user-centric development, are of the utmost importance.

At present, several wearable companions have reached the market. Preliminary studies have aimed to evaluate how patients have implemented wearables in daily life, moving away from assessing hypothetical tolerability and toward the evaluation of hands-on device experiences. Major complaints have arisen such as the disapproval of bulky designs, the presence of wires and/or electrodes, and the necessity for adhesive material on the skin (124), whereas those devices deemed comfortable had secure fittings and discrete form factors (122). In general, patients are “device-naïve” insomuch as they have not previously used wearables. Thus, an uncertainty exists as to whether user-error could impact the implementation of a seizure detection strategy, and consequently hinder further development efforts. Depending on a device's user interaction, its manipulation may require sufficiently preserved executive function in order to learn a sequence of procedures, such as pairing a sensor bracelet to a mobile application. Furthermore, rapidly adapting to the device when prompted, such as charging or even changing the battery, could create obstacles for efficient use. Recently, a study examining a wrist-worn device demonstrated that only 50% of patients were able to fully and independently control it, whilst others needed both appropriate support and training, and a subgroup of patients (13.3%) required constant supervision (123). In several devices, users have identified constraints early on. For example, a phase 2 study showed that 14% of patients consistently misplaced the EMG patch (93), whereas another lost 54% of the patient samples due to mishandling and data connection failures (100). Still others found possible handling issues in real-world conditions, leading to a 10% loss in device users during a phase 4 study (32).

Roadblocks such as these will specifically deter patients with high seizure frequency rates, as cognitive impairment is increased in the majority of these cases (125). In essence, difficulties in usability risk hinder the effectiveness of these detection and forecasting devices in patients with very active epilepsy, a population subset which should be the ones who benefit most from wearables with alarms.

## Conclusion

Mobile health devices show promise for patients suffering from epileptic seizures. However, before they can be widely proposed, and/or medical insurance coverage can be assured, several key efforts need to be solidified. When following state-of-the-art research (92) and clinical practice guidelines (10, 11), the overall recommendation is to perform robustly designed clinical validation studies in EMUs, in addition to real-life environment situations, to concretely demonstrate the reliability of GTCS detection and quantification algorithms, as well as other seizures types. Especially, since currently, mobile health devices have only shown validity for the detection of GTCS, and not for other seizure types. Furthermore, clinical outcomes need to be assessed, including the decreased morbidity and mortality associated with seizures and improvements in quality of life. The future of wearable mobile health devices related to epileptic seizure detection or forecasting must continue to focus on the advancement of adequate sensors, and their development should emphasize user-centric methods prior to products entering beta testing. At present, there is still a gap between what seizure detection devices are capable of measuring and the needs of patients. Specifically, prior to the implementation of mobile health companions into real-world situations, device developers should consider the clinical characteristics of the patients themselves and directly assess how digital health tools can directly benefit the management of epilepsy.

## Author Contributions

All authors listed have made a substantial, direct and intellectual contribution to the work, and approved it for publication.

## Funding

The review work and the publishing fees were funded by a grant from the Swiss National Science Foundation (SNF). Number: 320030_179240 Quantifying the severity of generalized tonic-clonic seizures (GTCS) with ambulatory connected devices (SEVERITY).

## Conflict of Interest

The authors declare that the research was conducted in the absence of any commercial or financial relationships that could be construed as a potential conflict of interest.

## Publisher's Note

All claims expressed in this article are solely those of the authors and do not necessarily represent those of their affiliated organizations, or those of the publisher, the editors and the reviewers. Any product that may be evaluated in this article, or claim that may be made by its manufacturer, is not guaranteed or endorsed by the publisher.
